# Targeted delivery of a short antimicrobial peptide (CM11) against *Helicobacter pylori* gastric infection using concanavalin A-coated chitosan nanoparticles

**DOI:** 10.1007/s10856-023-06748-w

**Published:** 2023-08-31

**Authors:** Mehrdad Moosazadeh Moghaddam, Shahin Bolouri, Reza Golmohammadi, Mahdi Fasihi-Ramandi, Mohammad Heiat, Reza Mirnejad

**Affiliations:** 1 Tissue Engineering and Regenerative Medicine Research Center, Baqiyatallah University of Medical Sciences, Tehran, Iran; 2 Research and Development Unit, Varia Hooman Kara Company, Tehran, Iran; 3 Baqiyatallah Research Center for Gastroenterology and Liver Diseases (BRCGL), Baqiyatallah University of Medical Sciences, Tehran, Iran; 4 Molecular Biology Research Center, Systems Biology and Poisonings Institute, Baqiyatallah University of Medical Sciences, Tehran, Iran

## Abstract

*Helicobacter pylori* is the cause of most cases of stomach ulcers and also causes some digestive cancers. The emergence and spread of antibiotic-resistant strains of *H. pylori* is one of the most important challenges in the treatment of its infections. The present study aims to develop a concanavalin A (ConA) coated chitosan (CS) nanocarrier-based drug delivery for the targeted release of peptides to the site of *H. pylori* infection. Accordingly, chitosan was used as an encapsulating agent for CM11 peptide delivery by applying ionotropic gelation method. Con-A was used for coating CS nanoparticles to target *H. pylori*. The CS NPs and ConA-CS NPs were characterized by FTIR, dynamic light scattering (DLS), and scanning electron microscopy (SEM). The MIC of CM11-loaded ConA-CS NPs against *H. pylori* SS1 strain was analyzed in vitro. In order to evaluate the treatment efficiency in vivo, a gastric infection model of *H. pylori SS1* strain was established in mice and histopathological studies and IL-1β cytokine assay were performed. Based on the results, the size frequency for CS NPs and ConA-CS NPs was about 200 and 350 nm, respectively. The prepared CM11-loaded ConA-CS NPs exhibited antibacterial activity against *H. pylori SS1* strain with a concentration of 32 µg/ml. The highest healing process was observed in synthesized CM11-loaded ConA-CS NPs treatments and a significant decrease in IL-1β was observed. Our findings highlight the potential of chitosan nanoparticles as a drug delivery vehicle in the treatment of gastric infection model of *H. pylori* SS1 strain.

**Graphical Abstract:**

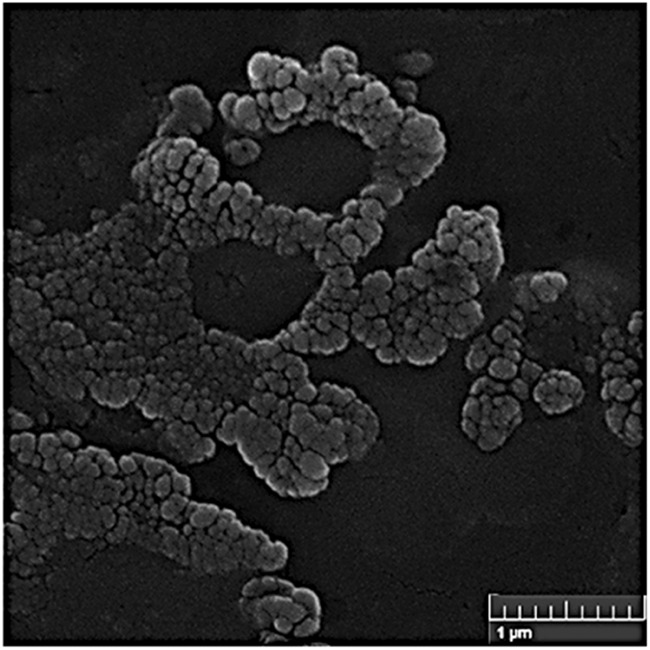

## Introduction

*Helicobacter pylori* (*H. pylori*) is a Gram-negative, motile, and microaerophilic bacillus, which causes worrisome digestive diseases such as acute and chronic gastritis, gastric ulcer, gastric cancer, gastric lymphoid malignancies, and also can cause some other diseases, especially diseases related to the liver [1, 2]. Generally, *H. pylori* is the most prevalent human gastric bacterial infection, colonizing more than 50% of humans, although only 10–20% of affected individuals will show clinical symptoms [3–5]. Effective treatment of *H. pylori* infection in patients suffering from acute clinical complications is an important strategy in preventing gastric cancer and MALT lymphoma [6, 7]. Antibiotic-based treatment is the main treatment method to deal with this bacterium. Various antibiotics such as amoxicillin, clarithromycin, metronidazole, levofloxacin, furazolidone, and tetracycline are effective against *H. pylori* infection [8]. This treatment regimen usually includes the simultaneous use of two antibiotics such as metronidazole and clarithromycin, which is considered as the first line of treatment. However, when this treatment is not successful due to antibiotic resistance, an alternative method is used, which includes the use of antibiotics such as metronidazole, and tetracycline along with omeprazole as a proton pump inhibitor antibiotic (PPIs) [9, 10]. According to the report of the World Health Organization (WHO), antibiotic resistance is one of the most important health challenges in the coming years dealing with microbial infections [11], and infection caused by antibiotic-resistant *H. pylori* is one of the most important parts of this concern [12, 13]. Therefore, in recent years, many studies have been conducted to provide new treatment strategies to deal with microbial infections. The use of antimicrobial peptides (AMP) is one of the most important strategies. Many studies have shown that these peptides are significantly effective against resistant bacterial strains and the processes of resistance against them occur very slowly [14, 15]. These peptides are part of the innate immune system in vertebrates and invertebrates and have been developed during the evolutionary process to deal with infectious agents. AMPs are usually cationic and amphipathic small molecules composed of less than 60 amino acids, which are effective against a wide range of Gram-positive and negative bacteria [14, 16]. In general, AMPs mostly target the bacteria’s membranes via interaction with negatively charged compounds in bacterial membranes forming various pores on the cell surface, which leads to the bacteria death [17]. Several studies have shown that cationic peptides have a significant potential to fight *H. pylori* [18–20]. CM11 peptide is a 11-residue sequence cationic peptide with and composed from C-terminal domain of melittin and N-terminal domain of cecropin A. Previous studies revealed that the CM11 peptide has significant antimicrobial activity against multi-drug resistant pathogens such as *Acinetobacter baumannii*, *Salmonella typhimurium*, *Staphylococcus aureus*, *Klebsiella pneumonia*, *Pseudomonas aeruginosa*, and *Escherichia coli* [21–23]. However, since cationic peptides such as CM11 do not act selectively against prokaryotic and eukaryotic cells, they can also be toxic against eukaryotic cells [15]. Studies have shown that the amount of negative charge on the surface of the membrane plays a key role in the affinity of the peptide to the membrane and its accumulation on the cell surface, and therefore more destructive activity [17]. Therefore, due to the greater negative charge of the membrane of bacterial cells compared to eukaryotic cells, the toxicity of the peptide against bacterial cells is higher [17, 22]. The entrapping of drugs such as AMPs in targeted nanocarriers is one of the studied methods that may reduce the dose and increase the effectiveness of the peptide for target cells and reduce its toxicity for other cells [24–26]. Chitosan is a suitable nanocarrier with extensive usage in different fields such as drug delivery systems due to its biocompatibility, biodegradability, and modifiability [27]. In addition, the naturalness of chitosan has beneficial properties including low toxicity, hemostatic properties, antimicrobial, antioxidant effects, and also immune-stimulating ability [28]. Additionally, some valuable features of chitosan are significantly important in its applications to humans, such as the ability to decrease inflammatory processes, especially in epithelial cell-based tissues [29]. For the targeted delivery of the drug encapsulated in the nanocarriers such as chitosan, it is necessary to modify the surface of the nanoparticle with agents that have a binding tendency to some ligands on the surface of the target cells [30, 31]. Accordingly, concanavalin A (ConA) as a lectin can be used to target *H. pylori* [32]. The pathogen’s ability to stick to mucosal surfaces and avoid elimination mechanisms such as peristalsis is very important for their colonization. It seems that the adhesion of *H. pylori* to the epithelial cells of the stomach is a prerequisite for the development and continuation of the infection [33]. The cell wall surface of *H. pylori* has glycoprotein-rich structures with significant mannose and fucose residues, which mediate the adhesion of the bacteria to specific mannose and fucose lectin receptors on the surface of gastric mucosal cells for colonization. Accordingly, concanavalin A is a lectin that is extracted from jack beans plant which can bind to the mannose residues of different glycoproteins [32, 34]. Therefore, it can be used to target nanoparticles against *H. pylori* bacteria.

Due to the importance of antibiotic resistance and the significant prevalence of antibiotic-resistant strains of *H. pylori*, which has challenged the treatment of infections caused by it, in the current study, we aimed to develop ConA-coated chitosan nanoparticles containing CM11 peptide to targeted delivery of the peptide into the *H. pylori* site of infection via immobilizing the CS chitosan nanoparticles with ConA because of its affinity to mannose residues of the *H. pylori* surface glycoproteins.

## Material and methods

### Peptide synthesis

The CM11 peptide was synthesized by GenScript USA, lnc; with approximately 98% HPLC purity.

### Bacterial strain and in vitro susceptibility assay

Amoxicillin and clarithromycin-resistant *H. pylori* SS1 strain was obtained from medical microbiology research center, Qazvin, Iran. *H. pylori* strain was cultured under microaerobic conditions, as previously published [4]. Briefly, the selected strain was spread on *H. pylori* agar plates enriched with 7% (v/v) horse blood, and culture plates were incubated at 37 °C for 24 h under a microaerobic condition (Anaeropack-Anaero, Mitsubishi, Japan). *H. pylori* growing cells in estimated exponential phase were suspended in sterile phosphate-buffered saline (PBS) (pH7.4), and adjusted to 0.05 OD with Brucella broth medium; the obtained suspension was poured onto a microtiter plate. CM11 antimicrobial peptide was prepared at different dilution (0 to 256 μg/ml) using sterile phosphate buffered saline (PBS). Antibiotic resistant *H. pylori* SS1 strain and antibiotic susceptible strain (ATCC 43504) were used for susceptibility testing. All culture plates were kept under microaerophilic atmosphere at 37 °C overnight. The minimum inhibitory concentration (MIC) and minimum bactericidal concentration (MBC) were determined as the minimum concentration of the drug that inhibits bacterial growth or is bactericidal, respectively.

Also, in order to evaluate the performance of the peptide and the drug release system in comparison with the conventional treatment of resistant strains of *H. pylori*, an triple antibiotics mixture including omeprazole as a proton pump inhibitor, metronidazole, and tetracycline [10], which is named as the triple antibiotic mixture therapy in the in vivo study, was used.

### Preparation of CM11-loaded chitosan nanoparticle

CM11 peptide was encapsulated using chitosan (CS) natural polymer with ionotropic gelation method based on our previous investigations [26]. Briefly, 1 g medium-molecular chitosan with deacetylation degree of 85% and molecular weight of 60–160 kDa (IranChitosan, Iran) powder was dissolved in 50 ml glacial acetic acid 1% with constant stirring to promote dissolution at room temperature overnight to reach a clear solution. Then, 1 ml suspension of CM11 peptide with twice the MIC of the peptide in vitro (32 µg/ml) in sterile distillated water was mixed with 1 ml of 1% chitosan solution (dissolved in acetic acid solution). Further, the resulting mixture was allowed to combine for additional 2 h. Also, tripolyphosphate polyanion (TPP) solution was prepared with dissolving 1 mg of TPP powder in 1 mL distilled water. TPP solution added to chitosan suspension dropwise using magnetic stirrer at 600 rpm at room temperature for 30 min to form the chitosan nanoparticles (CS NPs). The resulting cloudy solution, because of formed nanoparticles, separated and nanoparticles were isolated from the solution by centrifuging (18,000 rpm) at 10 °C for 30 min. To remove unreacted polymers and unloaded peptides, the nanoparticles were washed with sterile double-distilled water three times by centrifuging (18,000 rpm) at 10 °C for 10 min. The resulting supernatant was applied to calculate encapsulation efficiency (EE%), and nanoparticles were kept at 4 °C for further steps.

### Coating of CM11-loaded CS NPs with concanavalin A

Concanavalin A (Con-A) (500 mg/ml) was suspended in the 0.1 M MES (2-(N-morpholino)ethanesulfonic acid) buffer (pH 5.2), as a coupling buffer, mixed with CM11-loaded CS NPs and incubated at 26 °C for 24 h on a rotator. Then, the ConA immobilized CM11-loaded CS NPs were separated from the medium and washed three times with 0.1 M K_2_HPO_4_/KH_2_PO_4_ (pH 7.4) and stored in PBS. Coupling efficiencies were calculated by determining the initial and final (before and after immobilization) concentrations of the CM11-loaded chitosan nanoparticles within the medium using a UV spectrophotometer (Shimadzu, Japan) [35].

### Characterization of CS NPs and ConA-CS NPs

Fourier transform infrared spectroscopy (FTIR), scanning electron microscopy (SEM), and dynamic light scattering (DLS) analysis were performed to investigate the morphology and exact size of the CS NPs and ConA-CS NPs.

#### FTIR analysis

The FTIR was performed to confirm CS NPs coating with ConA. A certain amount of CS and ConA, and ConA-CS NPs powders were injected into the FTIR analyzer (BrukerOptik GmbH, Germany), respectively, using potassium bromide in 1:100 ratios. The spectrum was taken in the range of 400–4000 cm^−1^ with a resolution of 2 cm^−1^ and 200 times scanning using the attenuated total reflection (ATR) method.

#### Scanning electron microscopy

For SEM imaging, NPs were gold-sputtered to be observed under the scanning electron microscope (AIS2100; Seron Technology, Uiwang-si, Gyeonggi-do, South Korea) at an acceleration voltage of 15 kV.

#### Dynamic light scattering analysis

The particle size distribution and zeta potential of the nanoparticles were measured by DLS using a Zetasizer (Nano–ZS, Malvern Instruments Ltd., Worcestershire, UK). DLS measurements were performed at 25 °C with a scattering angle of 173.

### Measurement of encapsulation efficiency (EE%)

Isolated nanoparticles were used to determine the amount of EE% and the peptide in the medium was measured by UV visible spectrophotometer (CARY 100, Varian, California, USA) at λ_max_ = 278 nm. The EE% efficacy was investigated based on this formula:

$${\rm{EE}} \% ={\rm{Wt}}-{\rm{Ws}}/{\rm{Wt}}\times 100$$

In the presented formula, EE% represents the entrapment efficiency of nanoparticles, Wt shows the total amount of peptide used for the preparation of nanoparticles, and Ws reveals the amount of unloaded peptide present in the supernatant.

### In vitro peptide release at different pHs

Gastric environment as a niche of *H. pylori* is acidic; therefore, drug release in the acidic pH should be done in a controlled and appropriate manner. For investigating in vitro peptide release from CM11-loaded ConA-CS was dispersed in PBS with two different pHs (5 and 7.4), then the solution was transferred to a dialysis bag (MWCO 12 kDa). The dialysis bags containing CM11-loaded ConA-CS were kept in 30 ml of PBS at targeted pH and then were moved in bain-marie at 37 °C and shaking was performed constantly at 130 rpm. After a while, 1 ml of fresh PBS was combined with the same volume of release solution. The amount of CM11 that was released into dialysate was measured by UV-visible spectrophotometer and was quantified using the standard CM11 curve.

### Time-killing assay

Bacteria strain were suspended to 1 × 10^5^ CFU/ml in Brucella broth medium enriched with 10% FBS and incubated with CM11, CM11-loaded CS NPs, CM11-loaded ConA-CS NPs, clarithromycin and amoxicillin, as well as a triple mixture of omeprazole metronidazole, and tetracycline, respectively. At 0, 2, 4, 8, 12, 24, and 48 h, samples were used for bacterial counts. Time-killing curves were calculated by plotting mean colony counts (log10 CFU/ml) vs time.

### In vitro cell cytotoxicity assay

To determine the CM11 peptide, CM11-loaded CS NPs, and CM11-loaded Con-A-CS NPs cell cytotoxicity, MTT assay was performed using the commercial Cell Proliferation Kit I (Sigma-Aldrich, St. Louis, Missouri, USA) according to the producer’s protocol. Briefly, human gastric adenocarcinoma cell-line (AGS cells) at a density of 5 × 10^3^ cells per each well were seeded in 96-well plates and then each well was treated and incubated with 32 μg/ml concentrations of all three studied substances for 24 h. After 24 h, 10 μl of MTT reagent (3-(4,5-dimethylthiazol-2-yl)−2,5-diphenyltetrazolium bromide) was mixed with each well, and the treated cells were incubated at 37 °C for 4 h. Dimethyl sulfoxide (DMSO), a color developing component, was used to stop the process. Then, absorbance at 560 nm was examined using a microplate reader (ELx808, BioTek Instruments, Winooski, Vermont, USA). The percentage of cell viability was determined according to Eq. 2:

$${\rm{Cell\; viability}}( \% )=({\rm{absorbance\; of\; treated\; cells}}\times 100 \% )/{\rm{absorbance\; of\; untreated\; cells}}$$

### Animal model and treatment

4–6 weeks old C57BL/6 male mice (*n* = 35) were obtained from Pasteur institute, Tehran, Iran and kept at 22 ± 2 °C of room temperature, and humidity of 50 ± 5% with free access to water and food and under a 12 h light/dark cycle. In the first step, saturated sodium bicarbonate was administrated intragastric. After 30 min, each studied mouse was given 1 × 10^9^ CFU/ml of drug-resistant *H. pylori* SS1 in 0.3 ml sterile PBS on days 3, 5 and 7, respectively. The infection of drug-resistant *H. pylori* SS1 was allowed to develop for 3 weeks. All mice were randomly assigned to five treatment groups (*n* = 7), including control (without infection), infection model (without treatment), infection model treated with free CM11 peptide, CM11-loaded CS NPs (32 µg/ml peptide), CM11-loaded ConA-CS NPs (32 µg/ml peptide), and a triple therapy by omeprazole, metronidazole, and tetracycline (suggested treatment for resistant strains) [10], respectively. Mice of the triple antibiotic mixture treatment group were first administered a dose of 138 mg/kg of omeprazole, followed by a lag time of 30 min before administration of two other antibiotics (tetracycline 14.3 mg/kg and metronidazole 28.5 mg/kg) [10]. All mice were administered intragastrically of 100 µl solution of different nanoparticles through oral gavage once daily for a consecutive 3 weeks. After 48 h, studied groups were sacrificed and the mice stomachs were cut along the greater curvature, and obtained sections were rinsed with PBS, then sections were cut into three longitudinal sections with each section used for assessment of interleukin-1β (IL-1β) cytokine and hematoxylin-eosin (H&E) staining.

### Histopathological studies and IL-1β measurement

Samples were fixed in 10% formalin solution and routinely processed. Briefly, tissues were sectioned at 4 μm and stained with hematoxylin and eosin, and microscopically observed with a light microscope (Nikon, Japan). Initially, the thickness of the skin epithelium and inflammation (neutrophil infiltration) were estimated. The IL-1β level in skin homogenate was also determined by ELISA according to manufacturer’s instructions (KPG, Iran). The level of IL-1β was calculated regarding to standard curves of purified recombinant IL-1β at various dilutions.

### Statistical analysis

statistical analysis was performed using GraphPad Prism software version 8 (GraphPad Software, Inc., CA, USA). One-way ANOVA followed by Bonferroni’s post-hoc comparisons tests were used to analysis differences between groups. The analyzed data were representative of three independent experiments expressed as the mean ± standard deviation (SD). Differences were considered statistically significant when *P* < 0.05; *P < 0.05, **P < 0.01, ***P < 0.001, and ****P < 0.0001 at a significance threshold value of *p* < 0.05.

## Results

### Characterization of CM11-loaded CS and ConA-CS NPs

In the current study, CM11-loaded chitosan nanoparticles were successfully obtained adopting the ion gelation method as reported previously [26], which is an encapsulation process based on crosslinking between cationic chitosan and anionic TPP. ConA coating of CS nanoparticles was done using MES buffer and NPs analyzed by FTIR, SEM, and DLS.

#### FTIR

**Fig. 1:**
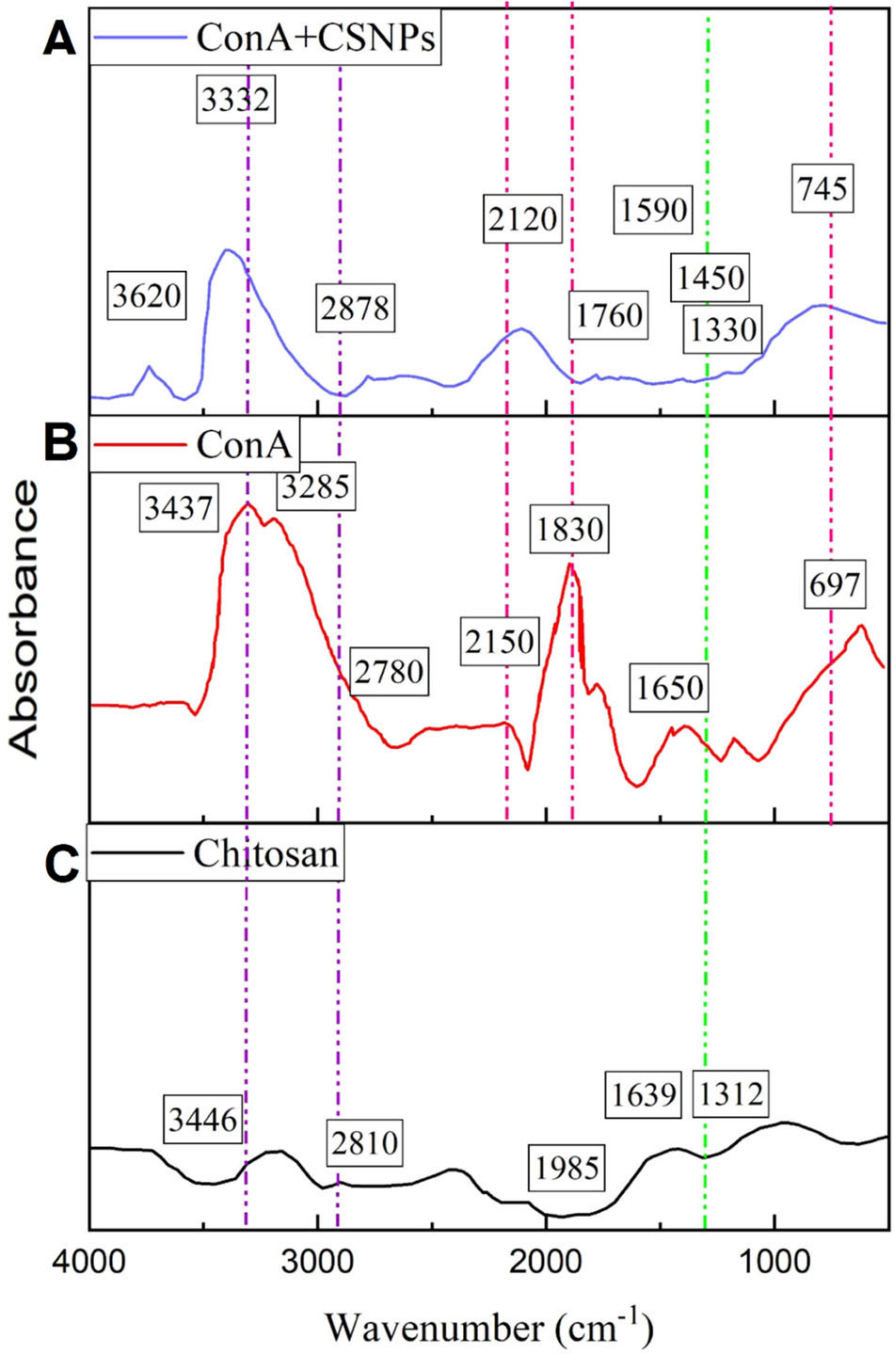
FTIR analysis of (**A**) ConA-CS NPs, (**B**) cancanavalin (**A**), and (**C**) chitosan samples. The spectrum was taken in the range of 400–4000 cm^−1^

FTIR analysis is a suitable method to determine the presence of organic compounds and the existence of functional groups and possible interactions between different compounds in the samples. Figure 1 shows the FTIR analysis for A) ConA-CS NPs sample, B) cancanavalin A sample, and C) chitosan sample. As can be seen in all three samples, the presence of O-H stretching bond in the range of 3400 cm^−1^ is related to water absorption in the samples. In the absorption spectrum of the chitosan sample (Fig. 1C), the broad peaks in the range of 3446 cm^−1^ are attributed to the stretching vibration of the water bond, hydroxyl groups, and amine groups. Also, the bond in the range of 2810 cm^−1^ is related to the asymmetric vibration of CH_3_ and CH_2_ in chitosan. The bond observed in the 2221 cm^−1^ range is related to the C–N bond of the C–NH_2_ group. The bond at 1655 cm^−1^ is observed for chitosan, it is related to the bending vibration of NH_2_, which indicates a high percentage of acetylation. For the ConA sample (Fig. 1B), absorption bonds are observed in the ranges of 3437, 3285, 2780, 2150, 1830, 1650 and 697 cm^−1^. The presence of absorption peak around 1385 cm^−1^ is related to C-O or C-H vibration and amide group. The absorption peak at 1650 cm^−1^ is related to the carbonyl structure. The presence of peaks at 3285 and 13,437 cm^−1^ is related to hydroxyl groups. In the ConA-CS NPs sample (Fig. 1A), all the peaks and links related to chitosan and ConA are clearly visible (745, 1335, 1450, 1590, 1760, 2120, 2878, 3332 and 3620 cm^−1^) and indicates the coating of CS NPs by ConA. The decrease in the intensity of the peaks in the ConA-CS NPs sample compared to ConA sample is due to the addition of chitosan to the structure, which acts as a chelator and takes the amine and hydroxyl groups of chitosan on the structure of ConA. Also, in the ConA-CS NPs sample, there is some peak shift compared to ConA, which is due to the electric charge transfer between the constituent components of the ConA-CS NPs and the local interaction between O, Co, and N with each other. In ConA-CS NPs sample, the presence of chitosan did not destroy the crystalline structure of ConA, and it shows that the structure of ConA is preserved.

#### SEM imaging

**Fig. 2:**
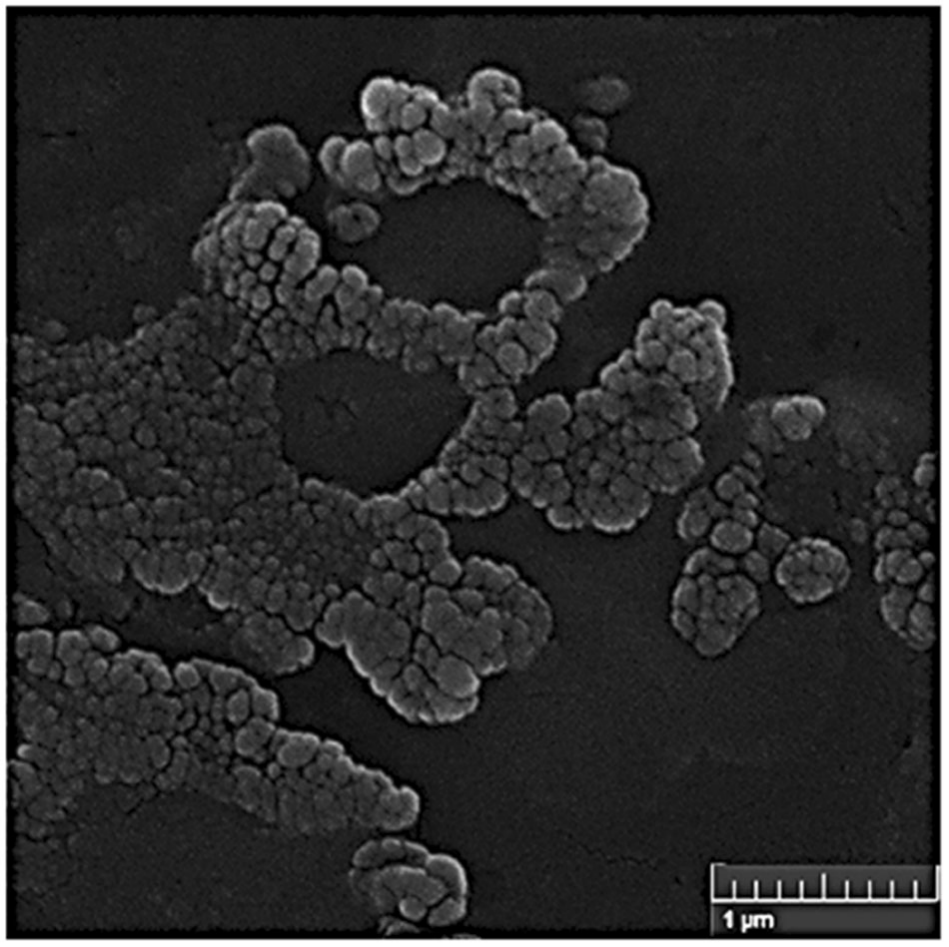
Scanning electron microscope photography of ConA-CS NPs

As seen SEM image in Fig. 2, the ConA-CS NPs were round in shape.

#### Particles size and zeta potential of NPs

**Fig. 3:**
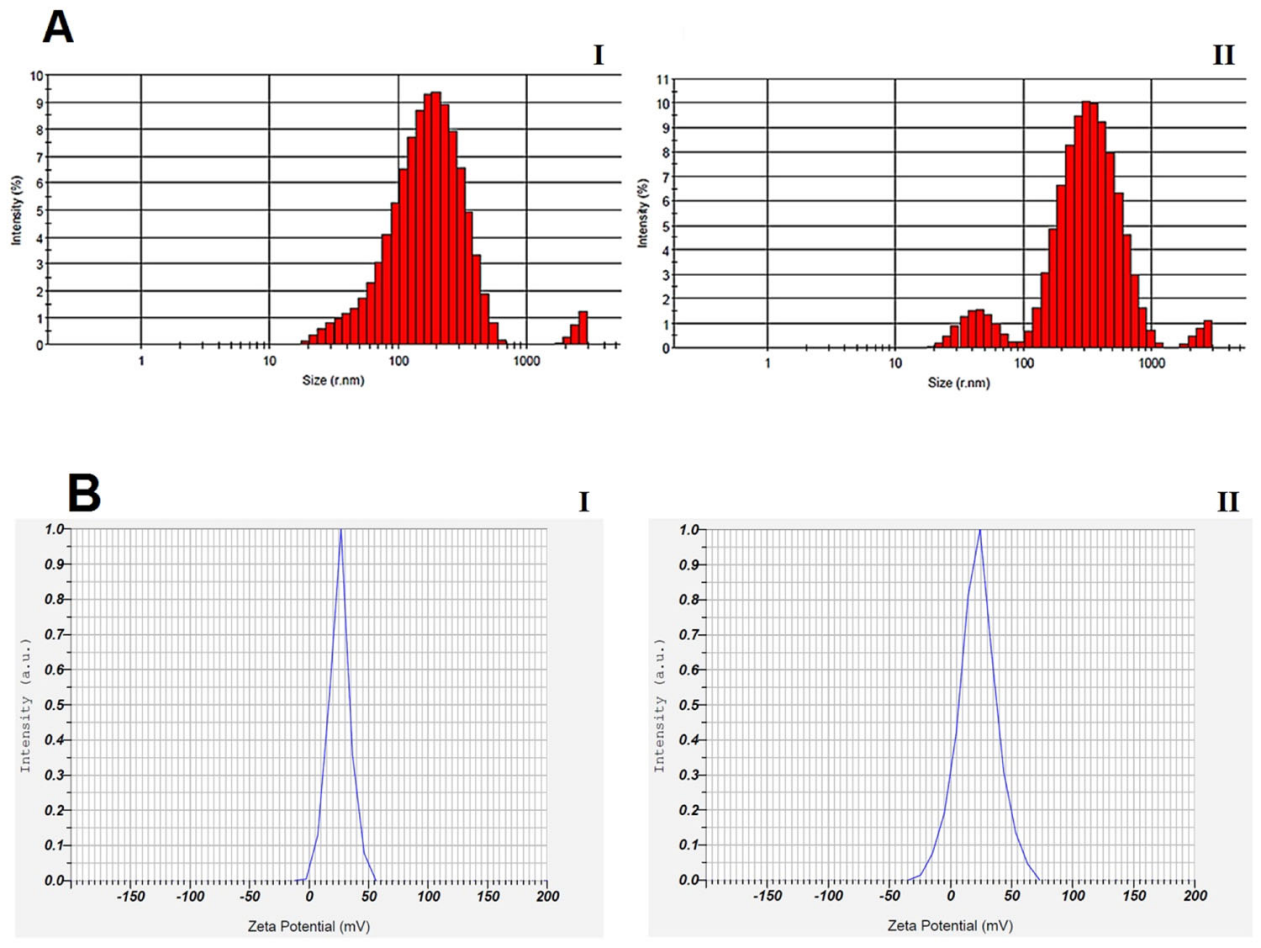
Diameter size (**A**) and zeta potential (**B**) of CS NPs (part I) and ConA-CS NPs (part II). Size frequency for CS NPs and ConA-CS NPs is about 200 nm and 350 nm, respectively. Zeta potential for CS NPs and ConA-CS NPs is 25.4 mV and 22.3 mV, respectively

Particles size and zeta potential of CS NPs and ConA-CS NPs shown in Fig. 3A, B. The DLS results showed particles size in the range of 60–600 nm with a size frequency of about 200 nm for CS NPs (Fig. 3A, part I) without crosslinking to ConA and 100–900 nm with a size frequency of about 350 nm for ConA-CS NPs (Fig. 3A, part II). The measure of zeta potential showed positive charges with 25.4 mV and 22.3 mV, respectively, for CS NPs (Fig. 3B, part I) and ConA-CS NPs (Fig. 3B, part II), which indicates a decrease in the surface charge of ConA-CS nanoparticles because of negative charge of ConA.

### Peptide release from NPs at different pHs

**Fig. 4:**
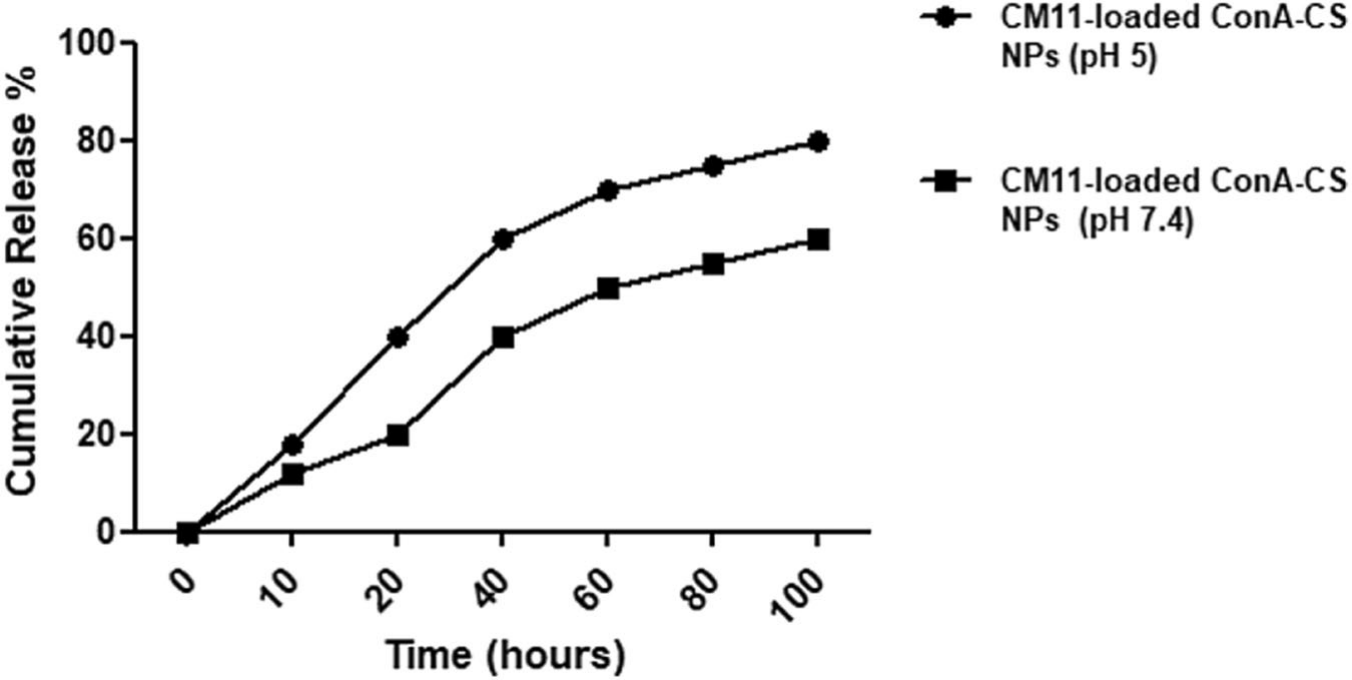
In vitro peptide release from CM11-loaded CS NPs and CM11-loaded ConA-CS NPs at two different pHs (5 and 7.4)

The bioavailability of the drug in gastric is significantly dependent on acidic pH (5–6). So, designing acidic pH-competent nanoparticles in comparison of physiological pH (7.4) may have potent activity against gastric pathogens. For this purpose, peptide release from CM11-loaded ConA-CS NPs was investigated at pH 7.4 and 5, which shown in Fig. 4. At the pH 5, majority of the entrapped peptide was released from CS ConA–CS NPs. According to the analyzes, peptide release from ConA–CS NPs was 60% at pH 7.4 while in the acidic environment was 85%.

### Anti-H. pylori activity of free CM11 peptide and CM11-loaded NPs using time-killing assay

**Fig. 5:**
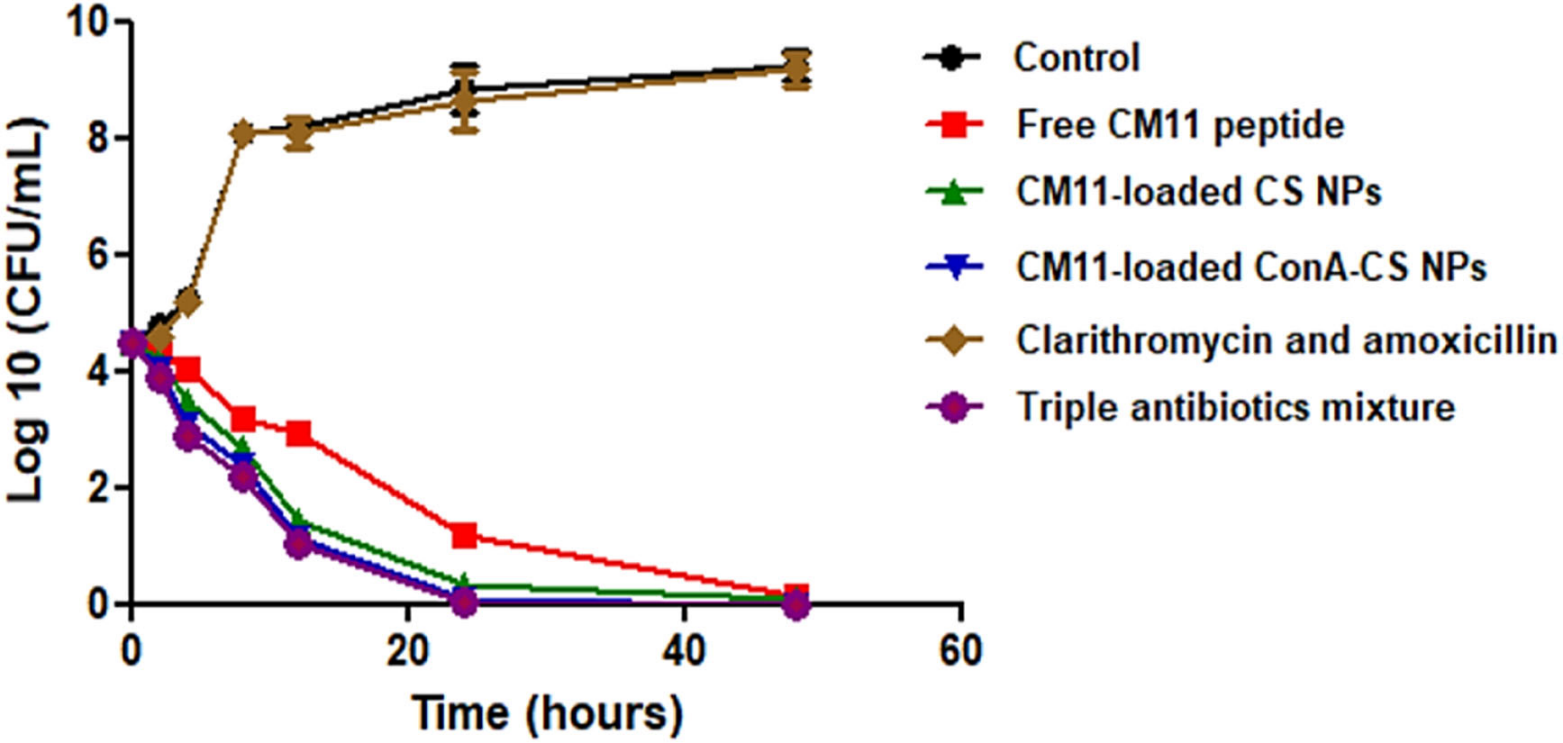
The Time-kill assay of free CM11, CM11-loaded CS NPs, CM11-loaded ConA-CS NPs, clarithromycin and amoxicillin, and triple antibiotics mixture (omeprazole, metronidazole, and tetracycline) against amoxicillin and clarithromycin-resistant *H. pylori* SS1 strain

The MIC of the free CM11 peptide against drug-resistant *H. pylori* SS1 strain was 16 μg/ml while drug-resistant *H. pylori* SS1 showed MICs and MBCs 64 and >128 μg/ml against clarithromycin and amoxicillin, respectively. To encapsulate the peptide in nanoparticles, twice the MIC concentration of the peptide (32 µg/ml) was used. The time-killing results showed that peptide-based and triple antibiotics mixture treatments compared to the untreated control group as well as treatment with two antibiotics, clarithromycin and amoxicillin, significantly reduce the bacterial growth in the investigated time period (*P* < 0.05). Accordingly, CM11-loaded ConA-CS NPs has a higher antibacterial potential compared to the CM11-loaded CS NPs and free CM11 peptide, respectively. While there was no significant difference between the treatment with CM11-loaded ConA-CS NPs and triple antibiotics mixture, which indicates that it has the same effect similar to triple antibiotic therapy. Time-kill curves showed that compared to the free peptide, CM11-loaded ConA-CS NPs and CM11-loaded CS NPs significantly reduced drug-resistant *H. pylori* SS1 after 12 h while amoxicillin and clarithromycin had no killing effect and their growth trend was same as the control group. The CM11-loaded ConA-CS NPs and triple antibiotics mixture were also able to kill drug-resistant *H. pylori* SS1 within 24 h while for free peptide and CM11-loaded CS NPs was after 48 h (Fig. 5).

### Effects of free CM11 peptide and CM11-loaded NPs on AGS cell viability

**Fig. 6:**
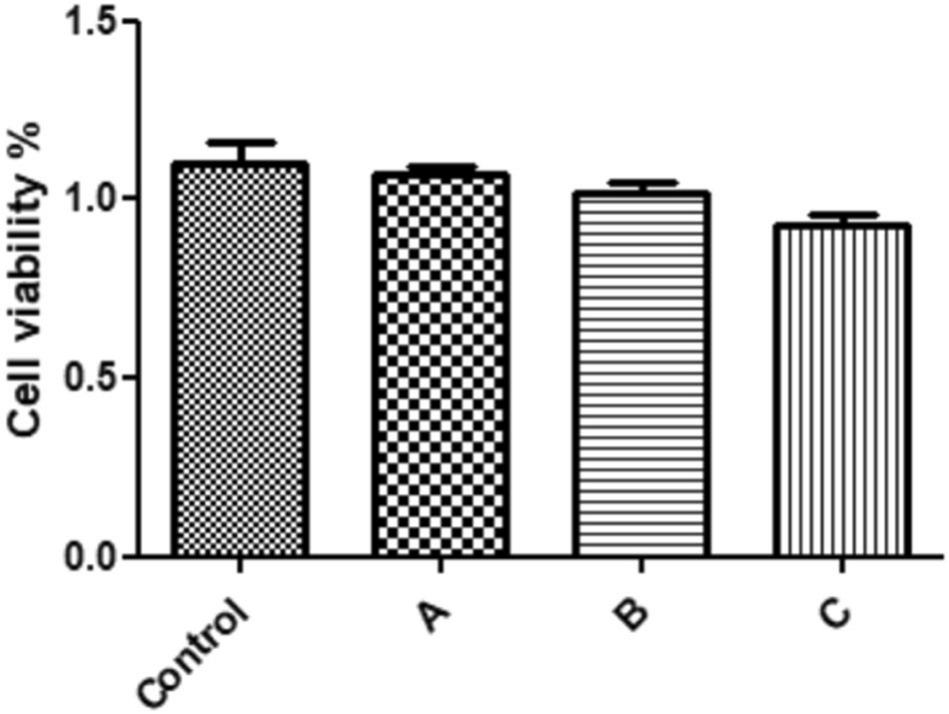
Cell viability assay of human gastric adenocarcinoma cell-line (AGS cells) treated with free peptide, CM11-loaded CS NPs, and CM11-loaded ConA-CS NPs. A) Effects of 32 μg/ml concentration of the CM11 antimicrobial peptide on AGS cells viability (free peptide); B) Effects of 32 μg/ml concentration of the CM11 peptide encapsulated in chitosan nanoparticles on AGS cells viability; C) Effects of 32 μg/ml concentration of the CM11 peptide encapsulated in ConA-coated chitosan nanoparticles on AGS cells viability. Data are shown as the mean ± SEM

To assess cytotoxic effects of free CM11 peptide, CM11-loaded CS NPs, and CM11-loaded ConA-CS NPs on AGS cells, direct microscopic examination and cell viability assay using MTT was performed. As shown in Fig. 6, compared with the control cells after 24 h, all the examined groups showed no adverse effects on AGS cell line. Accordingly, CM11-loaded ConA-CS NPs with 32 μg/m concentration of peptide were used in further in vivo study.

### Treatment of infected mice with CM11 peptide and CM11-loaded NPs

**Fig. 7:**
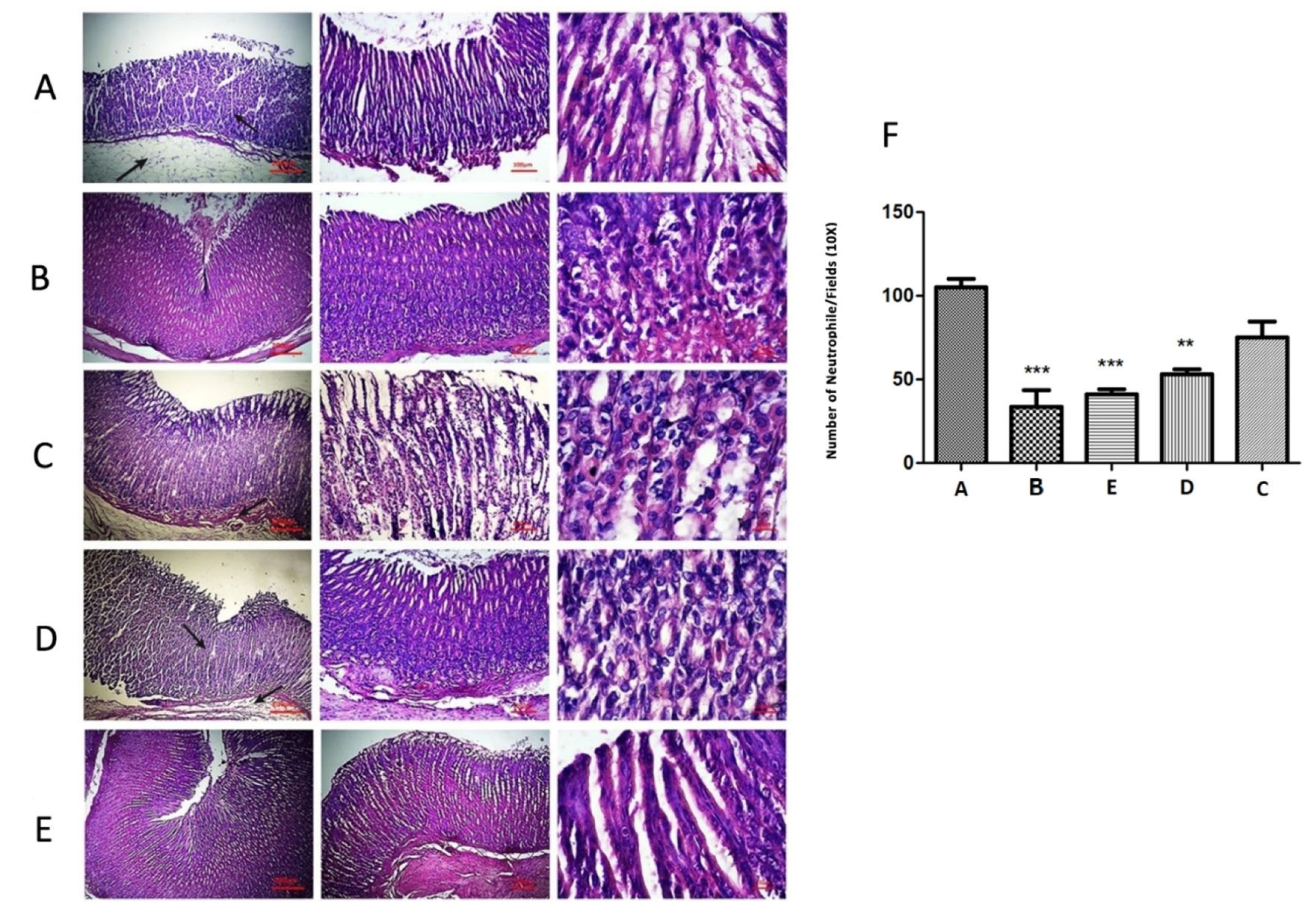
Histopathology graph comparing different treated groups. **A** Infection without treatment showing high neutrophil infiltration as a marker for inflammation and disrupted epithelium cells; (**B**) Infection with triple antibiotic mixture treatment revealed lower inflammation with normal epithelium appearance; (**C**) Infection treated with CM11 antimicrobial peptide (free peptide) indicates neutrophil infiltration and epithelia disruption; (**D**) Infection treated with CM11-loaded chitosan nanoparticles revealed the inflammation with neutrophile accumulation. **E** Infection treated with CM11-loaded ConA-chitosan nanoparticles revealed less inflammation with normal epithelium appearance compared to other groups; (**F**) Number of neutrophils in each microscope field (10X). Data are shown as the mean ± SEM. **P < 0.01, ***P < 0.001 by post hoc one-way ANOVA statistical analysis

**Fig. 8:**
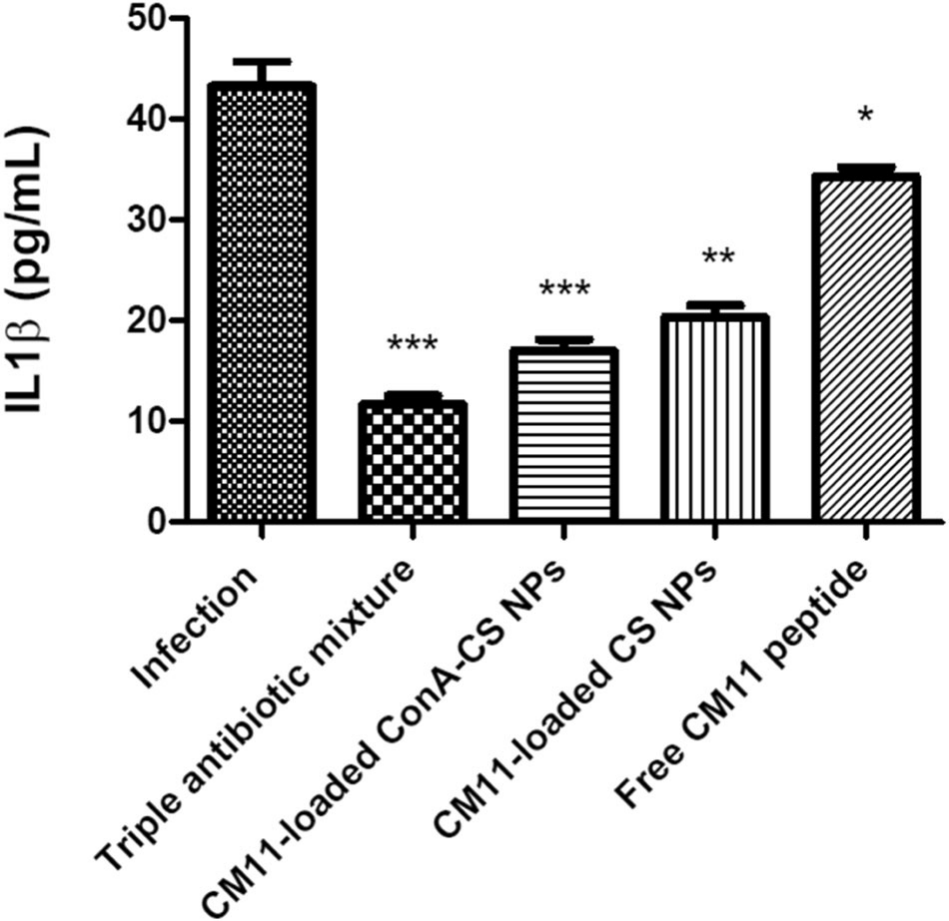
IL-1β assay in gastric biopsies from different groups reveals inflammation. IL-1β level significantly decreased in the triple antibiotic mixture treatment group and CM11-loaded ConA-CS NPs treatment. Data are shown as the mean ± SEM. *P < 0.05, **P < 0.01, ***P < 0.001 by post hoc one-way ANOVA statistical analysis

Based on the significant in vitro observations, mouse infection model was used to investigate the in vivo therapeutic efficacy of CM11-loaded ConA-CS NPs against *H. pylori* SS1 infection. Compared with untreated mouse gastric sections (control group), inflammation in the peptide and triple antibiotics mixture treated groups was significantly reduced. However, this suppression was more significant in the CM11-loaded ConA-CS NPs treated group compared to free CM11 peptide and CM11-loaded CS NPs groups. According to the obtained results from gastric sections, encapsulating the peptide in nanoparticles increases its effectiveness compared to the free peptide. On the other hand, coating the nanoparticle with ConA has also led to a further increase in this effect. Also, the penetration of a large number of inflammatory cells towards the stomach parts of the untreated models was visible while their number was decreased in free CM11 peptide and CM11-loaded CS NPs groups and the greatest decrease was seen in CM11-loaded ConA-CS NPs treated group (Fig. 7). In addition, the results of the groups treated with a mixture of antibiotics and CM11-loaded ConA-CS NPs were almost similar. The data obtained above demonstrated that CM11-loaded ConA-CS NPs can improve infectious diseases by effectively eliminating drug-resistant bacteria in a mouse gastric infection model. Also, free CM11 peptide and CM11-loaded CS NPs showed effective response but less than CM11-loaded ConA-CS NPs. Moreover, after twenty-one days of treatment, a significant decrease in IL-1β was observed in peptide-based treated groups compared to the untreated group. In peptide-based treatment, ELISA results showed a significant anti-inflammatory effect of CM11-loaded ConA-CS NPs (*P* < 0.001), CM11-loaded CS NPs (*P* < 0.01), and free peptide (*P* < 0.05) compared to the control, respectively, through a significant reduction of IL1-β levels. Among the peptide-based treated groups, the greatest decrease of IL1-β was observed in infected mice treated with CM11-loaded ConA-CS NPs (Fig. 8). However, among the groups, triple antibiotics therapy as triple antibiotic mixture treatment showed the lowest decrease in IL1-β, which was not significantly different from the CM11-loaded ConA-CS NP therapy.

## Discussion

*H. pylori* is a well-studied and concerning pathogen in various gastric disorders such as acute and chronic gastritis, peptic ulcer disease and gastric cancer and extra-gastroduodenal including liver diseases, cardiovascular diseases, and autoimmune disorders [36, 37]. As previously mentioned, over 50% of the world’s population carried *H. pylori* on stomach; therefore, paying attention to the prevention and treatment of infections caused by this bacterium is of particular importance [38, 39]. Currently, drug resistance of microbial pathogens is a global health-threatening [40] and, the success of the treatment is reduced by 60–70% due to the bacterial resistance [41]. This resistance is caused by various factors, including insufficient duration of treatment, premature discontinuation of the antibiotic and incorrect selection of the antibiotic [42]. Accordingly, the prevalence of antibiotic resistance in *H. pylori* is currently increasing worldwide and, like other pathogens, has become a growing public health problem that requires more attention [43]. In the treatment of *Helicobacter* infection, metronidazole and clarithromycin are used as first-line antibiotics, but in recent years, strains resistant to these antibiotics have expanded significantly, which has led to an increase in treatment failure, so choosing the appropriate antibiotic or producing new drugs to treat infections caused by this bacterium is necessary and unavoidable [44]. Antimicrobial peptides are among the remarkable compounds that have received much attention in recent years. Many studies have shown that these peptides are effective on a wide range of gram-negative and positive bacteria and resistance to them occurs at a very low rate [14, 45].

The present study aimed to evaluate the in vitro and in vivo potential therapeutic efficiency of CM11 antimicrobial peptide against clarithromycin and amoxicillin-resistant *H. pylori* SS1 strain. The peptide was encapsulated into chitosan nanoparticles coated with concanavalin A in order to release the peptide in a controlled and targeted manner and to help the stability of the peptide in acidic conditions of the stomach. At first, MIC-based susceptibility assay was used to demonstrate that CM11 peptide is effective against antibiotic-resistant *H. pylori* strain. Similar to previous studies, it was shown that the CM11 peptide is also effective against antibiotic-resistant strain of this pathogen. The obtained MIC (16 µg/ml) was almost similar to the effective dose of this peptide on other human pathogens that we have previously studied [21–23, 46, 47]. In this study, chitosan nanoparticles were used as nanocarrier. In many studies, chitosan has been developed and used as a valuable polymer, particularly in terms of biomedical applications such as drug delivery [48]. Transferring the drug to the gastrointestinal tract, such as the stomach through the mouth is one of the challenges of drug delivery. Several physiological barriers such as a highly acidic environment, degrading enzymes, and short residence time in the digestive system can lead to ineffectiveness or a decrease in the effectiveness of the drug. For this reason, the treatment can be affected by the incomplete release of the drug, which can be compensated by using higher doses of the drug but it is not a proper solution. For this reason, in recent years, the use of nanocarriers such as chitosan, which can protect drug from degradation and improve their targeted release has received much attention [49]. In addition to the various features of chitosan that were mentioned earlier, one of the unique features of chitosan is its antacid activity, which prevents the reduction of the effect of drugs in the stomach. Chitosan is a basic polymer and easily dissolves in acidic solutions, so this polymer dissolved by stomach acid and becomes a viscous gel that can protect its surrounding environment from acid. It also has amino groups that lead to H^+^ neutralization. This property leads to the emergence of conditions that can protect the drug from stomach acid [50, 51]. In general, drug release from CS NPs is more pH-dependent. In acidic pH compared to normal pH, the electrostatic attraction between protonated amine groups and H_2_O molecules is more significant, which leads to the dissolution of CS polymers and as a result of erosion of the NPs matrix, faster drug release occurs [51]. According to the acidic environment of the stomach, peptide release from CS nanoparticles was also evaluated in acidic (pH 5) and normal (pH 7.2) pHs. Based on findings, peptide release from ConA–CS NPs at pHs 7.4 and 5 was 60% and 85%, respectively, which is in accordance with the description provided. It should be noted that ConA tends to adhere to positively charged surfaces such as CS via electrostatic interaction, which is due to ConA’s overall negative charge under physiological conditions. Despite the negative charge of ConA, our analyses showed that the surface charge of the coated CS nanoparticles is still positive (22.3 mV), which indicates the suitable conditions of the NPs for use in an acidic environment. However, the isoelectric point (pI) of ConA is ∼5, meaning that it has no net electrical charge at the acidic pH of the stomach [52].

Characterization of the size of CS NPs shows that the size of ConA-coated CS NPs has increased from ∼200 nm to about ∼350 nm, which confirms the surface modification of CS nanoparticles by ConA. This modification was also confirmed by FTIR analysis, so for all the peaks and links related to chitosan and ConA were clearly visible in ConA-CS NPs which indicates ConA coating of CS NPs. In addition, the EE% of peptide was around 70%. These results are consistent with a similar study that we used hyaluronic acid-coated CS NPs to target delivery of CM11 peptide against CD44-overexpressing tumor cells. In the previous study, the EE% was 60%, which is almost similar to the results of this study. Also, the coating of nanoparticles with hyaluronic acid (HA) showed an increase in the size of CS nanoparticles. In addition, the coating of nanoparticles with hyaluronic acid showed an increase in the size of nanoparticles. Although hyaluronic acid has a negative surface charge, the overall surface charge of HA-coated CS nanoparticles was positive, which was also observed in this study for ConA-coated CS nanoparticles [26].

As it mentioned, in this study, we used ConA to target peptide-containing CS nanoparticles against *H. pylori*. ConA, as a lectin, has tetramer structure at physiological pH (7.4) composed from four identical monomeric subunits (MW ∼25 kDa). Each monomer monomeric subunit includes an independent carbohydrate binding site to which mannose and glucose monosaccharides can bind [52]. Since the membrane of *H. pylori* has glycoproteins rich in mannose, therefore, ConA was used to target CS nanoparticles. Accordingly, time-killing results showed that compared to free CM11 peptide and uncoated CS NPs, ConA-coated CS NPs are more effective against *H. pylori*. In the first 12 h, free peptide and CM11-loaded CS NPs led to a reduction of 2 and 3 log of bacteria, respectively, while CM11-loaded ConA-CS NPs reduced 4 log of bacteria compared to control. In addition, after 24 h, the bacteria treated with the CM11-loaded ConA-CS NPs were killed while this happened after 48 h in the case of the other two groups. Based on results of time-killing, there is a significant difference between the free peptide and the CM11-loaded ConA-CS NP activity against *H. pylori* while the performance of the uncoated CS NP was close to the ConA-coated CS NPs. It is assumed that the positive charge of chitosan nanoparticles (amino groups of glucosamine) can lead to the accumulation of these particles on the surface of the bacterial membrane (negatively charged) [53], and therefore its antibacterial effect is greater than the free peptide. On the other hand, the results showed that the performance of the peptide is comparable to the treatment based on the triple antibiotic mixture, which is proposed to treat the infection caused by the antibiotic-resistant strains.

According to these results, we evaluated the effect of peptide-loaded nanoparticles in an animal model with *H. pylori* infection. *H. pylori* infection can cause inflammation in the gastric area. Its infection is characterized by the infiltration of inflammatory cells into different areas of the infection, which results in superficial damage to the surface epithelium of the stomach tissue [36, 37]. In the present study, according to the hematoxylin and eosin staining results, we showed that inflammatory responses and accumulation of bacteria in gastric were significantly decreased in group that treated with CM11-loaded ConA-CS NPs and triple antibiotics mixture, respectively. This result highlighted the potent therapeutic effects of CM11-loaded ConA-CS NPs compared with other treatments including CM11-loaded CS NPs and free peptide. IL-1β is one of the best-studied mediators of *Helicobacter*-induced gastritis and upregulation of IL-1β is pronounced in *H. pylori*-infected patients who manifest high levels of IL-1β in their gastric mucosa [38, 39]. Obtained results revealed that CM11-loaded ConA-CS NPs could diminish the expression of IL-1β in gastric secretions. These observations indicated that the CM11 antimicrobial peptide, which entrapped in ConA-coated chitosan could significantly inhibit the host inflammatory response caused by *H. pylori* infection and further alleviate gastric inflammatory injury.

Based on this, several similar studies have been reported. For example, Jain and Jangday developed a ConA conjugated gastroprotective multiarticulate delivery system of clarithromycin (CM) for the treatment of *H. pylori* [54]. They used ethylcellulose microspheres NPs as carrier for CM, which targeted via ConA. According to their report, adherence of ConA to the ethylcellulose microspheres highly elevated the muco-adhesiveness and also controls the delivery of clarithromycin in simulated gastric juice. They showed that created targeted delivery system may effectively eliminate the colonization of *H. pylori*. In a study conducted by Sngsantikul et al. [55] coating nanoparticles with gastric epithelial cell membrane were used to targeted delivery of the clarithromycin against *H. pylori* infection. In their study, gastric epithelial cells plasma membranes such as AGS cells were obtained and coated onto antibiotic-loaded polymeric structures. The obtained nanoparticles (as AGS-NPs) showed inherent sticking to *H. pylori* bacteria. It was shown that when AGS-NPs were incubated with *H. pylori* bacteria in vitro, they preferentially accumulated on the bacterial surfaces. Using in vivo study of *H. pylori* infection and clarithromycin as an antibiotic, clarithromycin-loaded AGS-NPs significantly showed therapeutic efficacy compared to the free drug as well as a non-targeted NP control group. The results of these studies are consistent with our findings and show that targeted release using polymeric nanoparticles is an efficient method to control *H. pylori* infections.

In this field, other similar studies have been conducted against some other bacterial pathogens. For example, Sobhani et al. [56], evaluated the antibacterial activity of CS nanoparticles and ciprofloxacin-loaded CS nanoparticles against *Escherichia coli* and *Staphylococcus aureus* by calculation of MIC. Results revealed that MIC of ciprofloxacin-loaded CS NPs was 50% lower than free ciprofloxacin in studied bacteria species. Similar to current study, CS NPs without drug exhibited antibacterial activity but at higher efficiency compared to free drugs. Moreover, Hassan et al. [57] used CS NPs as a carrier for tetracycline, gentamycin and ciprofloxacin delivery. Their nano-carriers with different antibiotics were reduced the growth of both Gram-negative and Gram-positive bacteria. In another study conducted by Taghipour et al. [26] to investigate targeted delivery of the CM11 peptide against some cancer cell lines with high expression of CD44 surface antigen using CM11-loaded HA-CS NPs, revealed that encapsulation efficiency of CM11 peptide in chitosan nanoparticles was approximately 60%. Same as our findings, their nanoparticles were pH-dependent, which peptide releasing was higher in acidic pH (5–6) compared with the neutral pH (7.4). Also, similar to above studies, CM11-loaded HA-CS NPs had higher toxicity against cancer cell lines compared to free peptide and CM11-loaded CS NPs.

## Conclusion

Overall, CM11-loaded ConA-CS NPs showed strong antibacterial activity against amoxicillin and clarithromycin-resistant *H. pylori* SS1 with an MIC of 32 µg/ml. Also, NPs revealed significant therapeutic potential against drug-resistant *H. pylori* SS1 infection for the elimination of the bacteria from gastric area of infected mice and the reduced inflammation in gastric sections of *H. pylori* infected mice. Our study demonstrates that CM11-loaded ConA-CS NPs have potential clinical application prospects and could be a strong suggestion for anti-infection therapy of *H. pylori*. However, this is an initial study that showed the remarkable performance of CM11-loaded ConA-CS NPs in an animal model, but more complete studies are needed to determine the safety and possible side effects, effectiveness and effective dose for use in the clinic.

## Data Availability

Data generated during the study are available from the corresponding author by request.
